# ArchPy and MODFLOW: Toward a General Integration of Heterogeneity into Groundwater Models

**DOI:** 10.1111/gwat.70028

**Published:** 2025-10-30

**Authors:** Ludovic Schorpp, Nina Egli, Julien Straubhaar, Philippe Renard

**Affiliations:** ^1^ Centre for Hydrogeology and Geothermics University of Neuchâtel, Neuchâtel Switzerland

## Abstract

Groundwater models are important and useful tools for answering scientific and technical questions about the quantity and quality of groundwater, as well as for making critical management decisions. However, the heterogeneity of subsurface properties, such as hydraulic conductivity, is known to play a central role in groundwater flow and transport; therefore, its accurate quantification and incorporation into the groundwater workflow are critical. This paper presents a novel tool, *ArchPy2Modflow*, that efficiently combines a stochastic geological generator, *ArchPy*, with a groundwater flow software, *MODFLOW*. *ArchPy2Modflow* provides a rapid and practical way to convert and link any ArchPy model to a new (or existing) MODFLOW model, where any *MODFLOW* spatial parameter (such as porosity, hydraulic conductivity, or storativity) can be obtained from an *ArchPy* property, which is then upscaled according to the *MODFLOW* grid. *ArchPy2Modflow* offers several different options for selecting the appropriate MODFLOW grid: using the same grid as in the ArchPy model, defining each ArchPy geological unit as a MODFLOW layer, coarsening the grid by a certain factor, or directly using an existing MODFLOW grid. This flexibility enables users to adapt their models to suit their needs and constraints. The usefulness and practicality of the new tool are demonstrated by a synthetic example considering flow and transport in a heterogeneous aquifer, while the impact of a particular grid selection on the simulations is demonstrated.

## Introduction

Groundwater models are one of, if not the most important, tools for answering scientific and technical questions regarding groundwater quantity and quality, and to take critical management decisions (Zhou and Li 2011; Doherty and Simmons 2013; Anderson et al. 2015; Miro et al. 2021; Schorpp et al. 2023). These models require the input of several parameters, generally related to the physical properties of the subsurface, which are known to be particularly heterogeneous depending on the geological context (Koltermann and Gorelick 1996; De Marsily et al. 2005). This heterogeneity is known to be strongly determined by geological history (Teles et al. 2004; Ringrose and Bentley 2016), and it has a significant impact on groundwater flow and transport due to the spatial variability of key physical properties, such as hydraulic conductivity, porosity, bulk density, or longitudinal and transverse dispersivities (Eaton 2006). Therefore, a proper characterization of the heterogeneity and the construction of an adequate geological model is crucial for accurate groundwater modeling.

Many software programs are available for groundwater modeling. Some of them are commercial (FEFLOW, Diersch 2013; GMS, HydroGeoSphere, Brunner and Simmons 2012). Others are freely available (MODFLOW, Langevin et al. 2017, 2022; OPENGEOSYS, Naumov et al. 2025; PARFLOW, Jones and Woodward 2001; MARTHE, Thiéry 1990). The MODFLOW suite is one of the most popular both in academia and industry thanks to its accessibility, its quality, and the strong support of the US Geological Survey. MODFLOW 6, its latest version, uses a control volume finite‐difference approach to solve the physical equations. It includes many different packages (e.g., dynamic river modeling or multilayered aquifer wells) and types of models (flow, multispecies transport, and heat), allowing a very flexible model construction. In addition, the Python programming interface FloPy (Bakker et al. 2016) has greatly facilitated the scripting of MODFLOW model creation and processing, including coupling with other software such as PEST++ (White et al. 2020; Schorpp et al. 2023).

On the heterogeneity modeling side, there is also a plethora of techniques and software available. The Texture2Par method (Scantlebury et al. 2025) is worth mentioning, as it can generate conductivity fields directly from lithological logs, without relying on complicated workflows. But usually, we can distinguish three main spatial scales at which geological heterogeneity is modeled.

First, structural geological models focus on the major (chrono‐)stratigraphic units. Structural models can be divided into two categories: explicit and implicit models (Wellmann and Caumon 2018). Explicit models directly represent the surfaces defining the boundaries between the units and the faults (Mallet 1992). Implicit models use one or several continuous latent variables defining level sets for the units (Calcagno et al. 2008; De la Varga et al. 2019; Grose et al. 2021).

The second scale of heterogeneity corresponds to the spatial distribution of lithologies or lithofacies within the stratigraphic units. Many methods exist to represent this heterogeneity (see reviews in Pyrcz and Deutsch 2014, Schorpp 2024), ranging from simple indicator simulations (Journel and Alabert 1989) to sophisticated process‐based physical models (e.g. Granjeon 1996).

Finally, the spatial variability of the petrophysical properties (e.g., porosity, hydraulic conductivity) within the lithologies or lithofacies corresponds to the finer scale of heterogeneity. Again, many methods such as geostatistics can be used to represent spatial variability at that scale (Matheron 1963; Chiles and Delfiner 2012).

These three scales of heterogeneity must generally be combined in a geological model through a hierarchical approach. With this principle in mind, the Python package ArchPy was developed (Schorpp et al. 2022) to propose an open‐source geological modeling toolbox. It automates and simplifies the process of constructing complex and hierarchical stochastic geological models.

The goal of this paper is to introduce an extension of ArchPy allowing its efficient linkage with MODFLOW. This extension can generate the MODFLOW grid from the ArchPy one or fill an existing grid with petrophysical parameters derived from the geological model.

When modeling heterogeneity, one must carefully consider the scale of the data used to constrain the models. For example, physical measurements of hydraulic conductivity (or porosity) are performed at a given scale (decimeters for a core sample in the laboratory, meters for a slug test, a few tens of meters for a pumping test, etc.). These measurements and their statistics (histogram, variogram, etc.) depend on the so‐called support or tested volume (e.g., Tidwell and Wilson 1997). Data collected on small supports have a broader statistical distribution (range of values) than data collected over a larger support volume. This is due to the averaging process that occurs when measuring data on a large support. Therefore, the heterogeneity model should be conducted at the same scale as the measurements; otherwise, it will not accurately represent the underlying physics. But if the support volumes are small compared to the area of interest, or if there is a need to use a grid with cells of various dimensions, one needs either to account for the change of support volume in the statistics (Mourlanette et al. 2020) or to make the heterogeneity model at the scale of the measurements and then upscale it to obtain a grid of reasonable size for the groundwater model. To provide a practical solution to this problem, we present in this paper an upscaling module included in ArchPy to manage, if needed, the scale discrepancy between the geological model and the flow model.

Upscaling a fine geological grid implies losing some information and details. Therefore, many different methods have been proposed to find a trade‐off between fast and accurate approximations. This topic is discussed at length in several review papers (Renard and De Marsily 1997; Sanchez‐Vila et al. 2006; Cetre‐Orejuela et al. 2024). The most accurate techniques imply solving local flow problems with a numerical method to determine the full tensor of hydraulic conductivity. For example, Zhou et al. (2010) provide a code following this approach. But in this paper, we implement faster methods based on real‐space renormalization (King 1989; Gautier and Nœtinger 1997; Renard et al. 2000). In particular, Renard et al. (2000) have shown that these techniques were faster than the numerical ones and that the loss of accuracy remained moderate.

The paper is organized in two main sections and a conclusion. The first section provides some background information about ArchPy and presents the new ArchPy2Modflow and Uppy modules. ArchPy2Modflow is an interface between ArchPy and FloPy. Uppy is a module that performs the upscaling required to transfer the information properly between the possibly different ArchPy and MODFLOW grids. The second section of the paper presents an application to illustrate the use of these new tools.

## Modules Descriptions

### ArchPy

ArchPy^1^ (Schorpp et al. 2022) is an open‐source Python package that produces geological models using a hierarchical approach. ArchPy uses three hierarchical levels: stratigraphic units, lithologies (or facies), and physical properties. Each level is simulated sequentially, constrained by previously simulated levels and existing geological data (geological map, boreholes, cross‐sections). The most important key feature of ArchPy is its Stratigraphic Pile (SP), which contains all existing expert knowledge and controls how the models are constructed. The SP includes, for example, the list of the different geological units to be simulated and their stratigraphic relationships, the lithologies these units contain, the geostatistical methods to be used, and their associated parameters. Hence, all the prior knowledge is contained in a single object, which can then serve as a basis for easier comparisons, for example, between different geological concepts. Another important advantage of ArchPy is that it can produce stochastic models, relying on different geostatistical approaches such as Gaussian Random Fields (GRFs, Matheron 1963, Chiles and Delfiner 2012), multiple‐point statistics (Strebelle 2002; Mariethoz et al. 2010), Sequential Indicator Simulations (SIS, Journel and Alabert 1989, Alabert and Massonnat 1990), or surface‐based approaches (Schorpp et al. 2025). Stochastic approaches allow for uncertainty quantification and also generate an ensemble of simulations that can be used with ensemble methods for model parameter identification (e.g., Neven et al. 2022; Neven and Renard 2023). The ArchPy's SP is also notably important as it allows easier comparisons of different geological concepts, where different SPs can be used and compared using either the cross‐validation procedure (Juda et al. 2020) or on other data such as groundwater data or geophysical data. The latter requires the use of forward models (e.g., MODFLOW) that use ArchPy outputs as input parameters.

More details and information on the construction and use of ArchPy can be found in Schorpp et al. (2022), Neven et al. (2022), Schorpp et al. (2024) and in the numerous tutorials available in the online documentation.^2^

### ArchPy2Modflow

ArchPy2Modflow is a new module implemented in ArchPy that facilitates the creation of MODFLOW models, starting from an existing ArchPy model. Currently, ArchPy2Modflow is compatible only with MODFLOW 6. It uses an object‐oriented programming approach in which an ArchPy2Modflow object is created from the base of an ArchPy Project (Arch_table object in Schorpp et al. 2022). In addition, ArchPy2Modflow creates the basic MODFLOW 6 modules with default settings (simulation object, steady‐state, simple solver, and a groundwater flow model). The core of ArchPy2Modflow lies in its ability to create a new MODFLOW grid based on an ArchPy grid. The *grid mode* parameter allows the user to decide how to upscale/downscale the ArchPy model to match the newly created MODFLOW grid. If necessary, ArchPy2Modflow can also perform geometrical operations to ensure a perfect alignment of the grids, such as grid rotation and grid origin matching.

**Figure 1:**
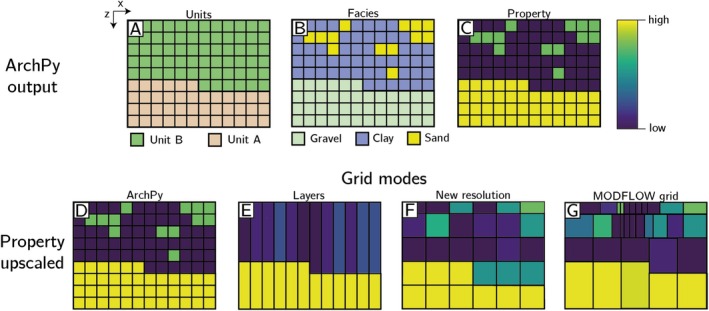
2D examples of available grid modes in ArchPy2Modflow using a schematic cross‐sectional view. (A–C) The ArchPy outputs in the original grid at the three hierarchical levels: units, facies, and property. (D–G) The resulting upscaled grids and properties according to different specified grid modes: ArchPy, Layers, New resolution, and MODFLOW grid. Detailed explanations about the various grid modes are given in the text.

Currently, ArchPy2Modflow offers four *grid modes*, which are illustrated in Figure 1. The top row (Figures 1A–1C) shows one schematic representation (2D vertical section) of a possible result from ArchPy: the stratigraphic units (Figure 1A), the lithologies or facies (Figure 1B), and the properties (Figure 1C).

In the second row (Figure 1D–1G), we see how the parameter values have been transferred to the MODFLOW grid using the different *grid modes*. Note that the information about the units and facies can be used in this process, but the main aim is to transfer the petrophysical parameters (porosity, hydraulic conductivity, etc.) to the MODFLOW grid.

- **ArchPy**. This mode is the simplest and consists in taking the same ArchPy grid for the ArchPy and MODFLOW models (Figure 1D).
- **Layers**. This mode creates at least n MODFLOW layers, where n is the number of ArchPy stratigraphical units (Figure 1A). The bottom elevations of these layers are derived directly from ArchPy simulations of the stratigraphic units, while the property values within the cells are averaged vertically (Figure 1E) using upscaling rules (which will be described later in this paper). Note that layers can be subdivided into a specified number of sub‐layers of equal thickness to better resolve vertical gradients where needed.
- **New resolution**. This mode upscales the ArchPy grid to a coarser resolution. The upscaling ratio is given as a specified factor in each direction. For example, Figure 1F shows a situation where a factor of 2 was used in all directions. It means that the number of cells is divided by 2 in each direction, so each 2 × 2 square of ArchPy cells is grouped and averaged to create a coarser MODFLOW cell. Different upscaling methods can be used to achieve this step. They are detailed in section 1.
- **Imported MODFLOW grid**. Instead of creating a new structured grid, the user can directly import a MODFLOW grid as an alternative *grid mode*. The grid can be structured or unstructured, but with some limitations. For now, the cells of the imported MODFLOW grid need to be exclusively rectangular cuboids. ArchPy2Modflow then takes care of identifying which cells from the ArchPy model fall in which cell of the MODFLOW grid and upscales them (Figure 1G) using Uppy. Concerning package compatibility, all MODFLOW 6 discretization packages can be used, such as grids from discretization by vertices (DISV) or unstructured discretization.

Although the ArchPy mode is the most accurate because it uses exactly the same resolution for both the geological and groundwater models, it may also imply numerical difficulties because of the possibly high number of cells, making the simulation computationally expensive and prone to convergence issues related to high parameter contrasts in nearby cells. Hence, the three other options offer the possibility to upscale the fine ArchPy grid to a coarser resolution. The Layers mode is the most extreme, as it may group a large number of cells, depending on the thickness of the units simulated with ArchPy. It is possible to mitigate this aspect by specifying the *lay_sep* parameter, which indicates the number of sub‐layers that must be used. When using this grid mode, it is important to check and ensure that the layers are well connected laterally. If this is not the case, numerical issues could arise from horizontal discontinuities within the layers. The New resolution grid mode offers slightly more control on the way cells are grouped and upscaled. Finally, the MODFLOW grid mode facilitates the importation of an existing MODFLOW model and the utilization of an ArchPy model to address heterogeneity in parameters such as hydraulic conductivity, porosity, and specific storage.

Most MODFLOW 6 packages whose parameters may vary locally can be handled by the current version of ArchPy2Modflow. These include the NPF (Node Property Flow) package for the groundwater flow model, the MIP (Model Input) and PRP (Particle Release Point) packages for particle tracking; the DSP (Dispersion), MST (Mobile Storage and Transfer) and ADV (Advection) packages for the transport model; the CND (Conduction and Dispersion) and EST (Energy Storage and Transfer) packages for the groundwater energy model. All the parameters for these packages can be informed using ArchPy property simulations.

### Uppy

Uppy is a collection of Python functions to upscale a physical property field defined on a fine regular grid towards a coarser, possibly unstructured, grid. For each coarse cell, Uppy determines the group of fine grid cells located within the coarse cell and computes an upscaled (or averaged) property at this new scale. Although it may be tempting to simply rely on the arithmetic mean for this task, this approach would be only valid for additive properties such as porosity (Matheron 1963; Renard and De Marsily 1997). For non‐additive properties, such as hydraulic conductivity, this approach is generally not applicable. Applying an arithmetic mean to the hydraulic conductivity would result in a systematic overestimation of the fluxes through the coarse model as compared to the fine‐scale model. It would also result in erroneous travel time estimations. A proper computation of the upscaled conductivity requires estimating the value of the equivalent conductivity tensor, which would ensure that the upscaled medium yields the same groundwater flux as the one that would be computed on the detailed model under the same head gradient.

A large number of upscaling techniques exist and have been reviewed (Renard and De Marsily 1997; Sanchez‐Vila et al. 2006; Zhou et al. 2010). To balance accuracy and computational efficiency, we have integrated only renormalization methods into Uppy, alongside standard mean functions (arithmetic, harmonic, and geometric). Renormalization methods generally provide a reasonable approximation of numerical upscaling while maintaining a moderate computational cost (Renard et al. 2000). Real space renormalization is an iterative process in which the fine grid is progressively upscaled through multiple intermediate resolutions, ultimately reaching the coarse grid. This process typically involves averaging small groups of adjacent cells using analytical expressions such as standard renormalization (King 1989), tensorial renormalization (Gautier and Nœtinger 1997), or simplified renormalization, which combines arithmetic and harmonic means (Renard et al. 2000). The averaging process is repeated until the target resolution is achieved.

Uppy integrates the three renormalization techniques: the standard, tensorial, and simplified renormalizations. The resolution change can be characterized by a reduction factor in each principal direction, indicating the extent to which the number of cells is reduced. For example, a reduction factor of 2 in the x direction and 3 in the y direction means that each group of fine cells of size 2×3 is merged into a single coarse cell.

**Figure 2:**
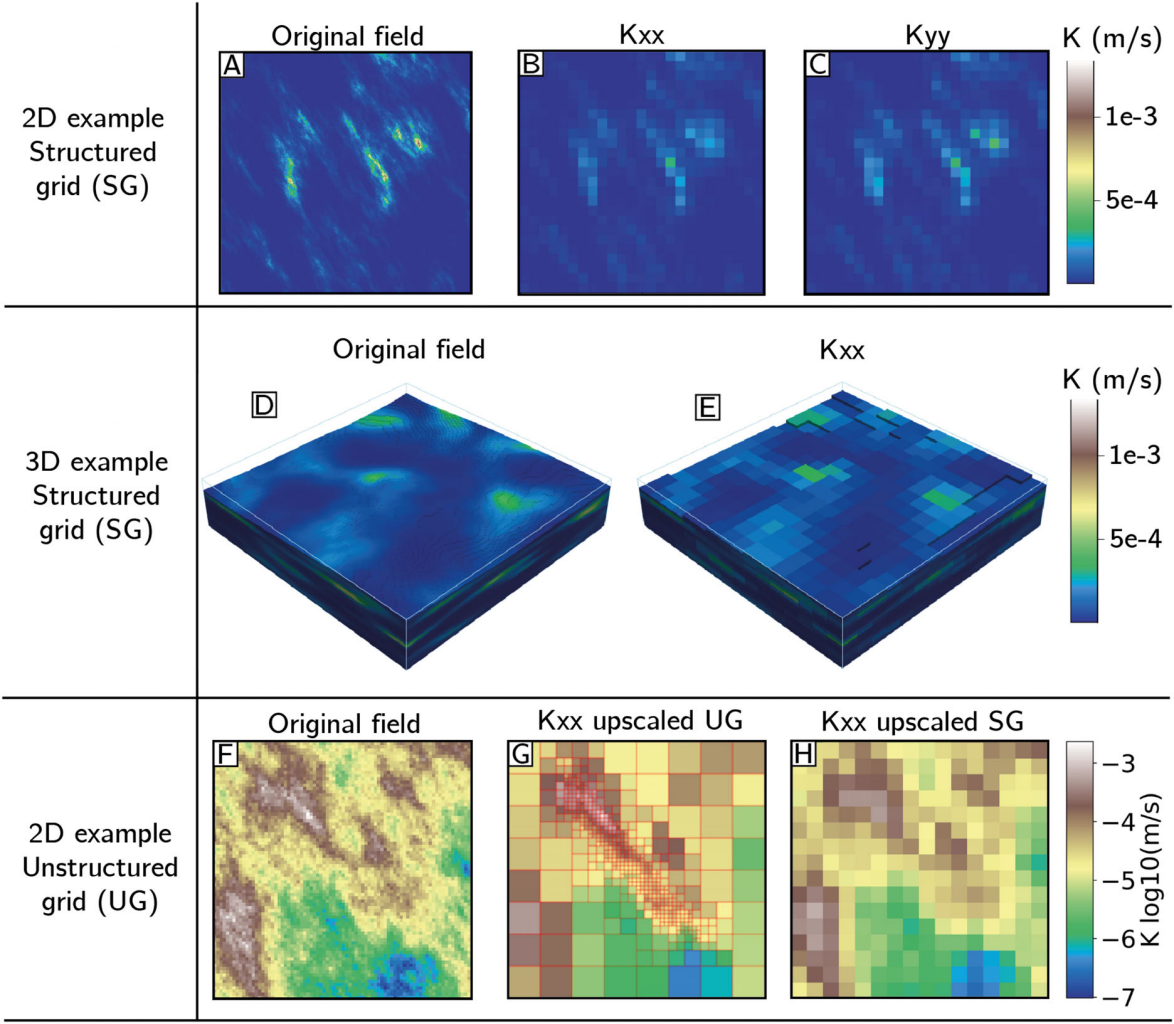
Illustration of the upscaling capacities of Uppy. The method used in these examples is the simplified renormalization method. (A) Original detailed 2D hydraulic conductivity (K) field. (B, C) Corresponding upscaled K field with a factor of 8 in both directions. The simplified renormalization method allows to obtain different upscaled value fields in the x, y and z directions. (D) Fine scale 3D K field. (E) Upscaled version of E with a factor of 8 in the three directions. The result is only shown in x direction. (F) Another 2D detailed K field. (G) Upscaled version of (F) on an unstructured grid. (H) Upscaled version of (F) on a regular structured grid for comparison.

**Figure 3:**
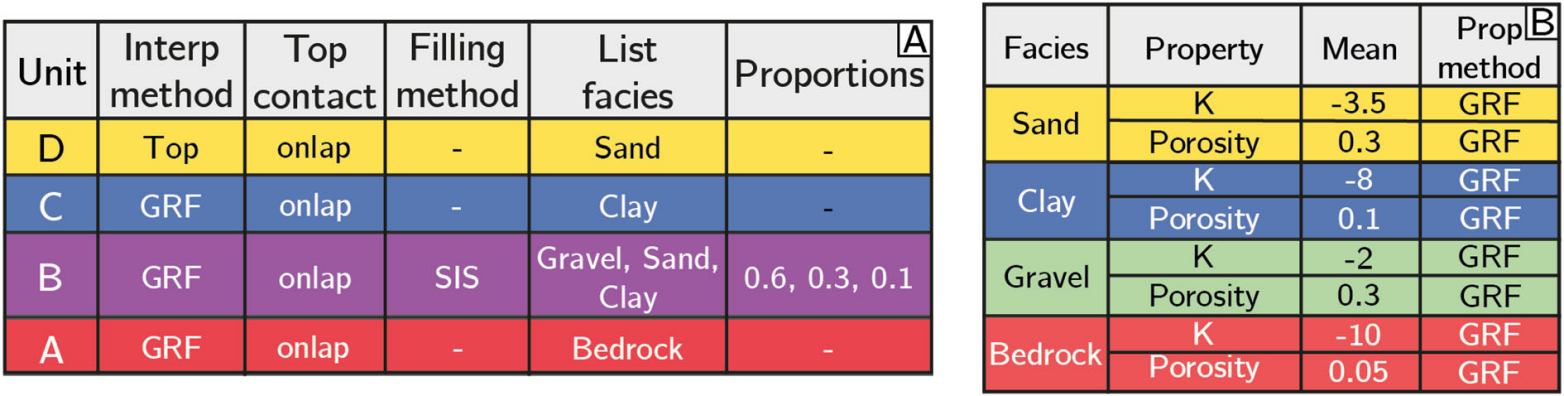
The example model's ArchPy Stratigraphic Pile. (A) Table containing the information required to simulate the surfaces delimitating the units (interp method and top contact) and the facies within the units (filling method, list of facies and proportions). (B) Table containing the information required to simulate the physical properties (property, mean, property [prop.] method) within the facies. The mean values for K are in $\log_{10}\left[\frac{m}{s}\right]$ units. The mean porosity is dimensionless. GRF, Gaussian random fields; Ho, homogeneous; *K*, hydraulic conductivity; SIS, sequential indicator simulation.

Figure 2 illustrates the main features of Uppy. It can handle the upscaling of 2D (Figure 2B, 2C, 2G, and 2H) and 3D (Figure 2E) grids. The only requirement for the original fields is that they are defined by a regular structured grid (Figure 2A, 2D, and 2F). Whatever renormalization method is used, Uppy outputs the upscaled value in the principal directions (x, y, and z if in 3D), which is particularly useful in groundwater modeling to represent anisotropy between the horizontal (x and y) and vertical (z) directions. In particular, when tensorial renormalization is used, Uppy directly outputs the full tensor, increasing the representativeness of the upscaling. Therefore, Uppy is able to partially preserve the finely simulated structures throughout the upscaling process. In addition, Uppy can handle some unstructured grids (UG), as shown in Figure 1G. For now, only UG that solely contain rectangular cuboids can be considered. Moreover, the only method that was implemented for such grids is the simplified renormalization and the simple averaging functions (arithmetic, harmonic, and geometric).

## Example of Application

To illustrate the new functionalities proposed in this paper, we consider the installation of a doublet (a combination of an injection well and a pumping well) in a heterogeneous aquifer. This situation is quite common in the case of geothermal exploitation or for groundwater treatment after a pollution event. This model aims to estimate the impact of the doublet on the groundwater system and to determine the capture area of the pumping well. With this example, we will demonstrate the capability of ArchPy2Modflow to upscale the fine model to a coarser model. Different upscaled models are proposed to show the differences between the available grid modes. In total, six models are constructed. Four models are obtained by upscaling the ArchPy model with the four grid modes: *ArchPy* and *Layers* with heterogeneity (Lay‐he), *New resolution*, and *MODFLOW grid*. In addition, two models, *ArchPy* and *Layers*, are tested with a completely homogenized geology. The code used to generate this example and the following figures can be found on the ArchPy GitHub.^3^

MODFLOW 6 is used to compute the groundwater flow and track particles. This is done using the PRT model, recently integrated into MODFLOW 6 (Morway et al. 2025). The PRT model directly calculates the advective particle trajectories in flowing groundwater based on the results of the groundwater flow model. It is capable of reproducing most of the capabilities of well‐known PRT software MODPATH 7 (Pollock 2016), such as handling structured and UG (using DISV package).

### Geological Model Settings

The extent of the modeled area is 150 m in the x‐direction, 75 m in the y‐direction, and 7.5 m in the z‐direction. The resolution is 1.5×1.5×0.15 m (sx×sy×sz) which implies a number of cells of 100 × 50 × 50 (nx×ny×nz). The top and bottom of the domain are assumed to be flat and set at −7.5 and −15 m, respectively.

The geological setting considers the presence of four hydro‐stratigraphic units from top to bottom:

- Unit D, relatively homogeneous thin (1 m) sandy aquifer.
- Unit C, thin (1 m) aquitard, mainly composed of clays.
- Unit B, main aquifer with a mean thickness of 3.5 m, composed of a mixture of gravels and sands with a minor fraction of clays.
- Unit A, bedrock, which is assumed to be completely impermeable and therefore defines the bottom of the main aquifer.

The corresponding ArchPy SP is shown in Figure 3. These tables detail the different geological objects to simulate, their stratigraphical and hierarchical relations (which unit is above which, which facies is present in which unit, etc.), and the different simulation methods to generate the model. The first simulation step of ArchPy involves simulating the top surface of each unit. In this example, GRFs are used for all the stratigraphic surfaces, except the first unit, where the surface is placed at the top of the model, as shown in Figure 3A. All the stratigraphic surfaces are considered depositional only (onlap type of contact).

The second simulation step involves facies modeling (filling method). We consider that most of the units are homogeneous as they only contain one facies (D, C, and A units). The unit B contains three facies (Gravel, Sand, Clay) simulated using the SIS method for its simplicity. Detailed variograms and modeling parameters are given in the Appendix.

The third and last step consists in populating the simulated facies with properties using continuous distributions. In this example, two petrophysical properties are required to solve the flow problem and compute travel times of particles: the hydraulic conductivity (K) and the porosity (n) (Figure 3B). The mean and variance of the distribution of these properties were chosen using typical values found in the literature (Domenico and Schwartz 1997). The SP defines which simulation methods must be used. Here, we use a 3D GRF to simulate $\log_{10}\left(K\right)$ to ensure that K has a lognormal distribution. The mean of $\log_{10}\left(K\right)$ is given in Figure 3B and its variance shown in Table A3. The simulation of the porosity is slightly different, because the porosity is bounded between 0 and 1. We therefore use the relation between porosity (n) and void ratio (e):

(1) $$n=\frac{e}{e+1}$$

The void ratio is a positive value between 0 and infinity. Like hydraulic conductivity, we can use a GRF to simulate the log10 of the void ratio. Then, we use Equation (1) to convert it to the porosity. The mean and variance of the logarithm of the void ratio were defined from the mean and variance of the porosity (Figure 3B) using a look‐up table of the correspondence between the mean/variance of the porosity and the mean/variance of the log10 void ratio. This table was constructed using a Monte Carlo procedure and directly implemented into ArchPy. The selected variance values are shown in the variogram parameters in Table A3. Borehole data are not included in these simulations.

### Detailed Geological Model

**Figure 4:**
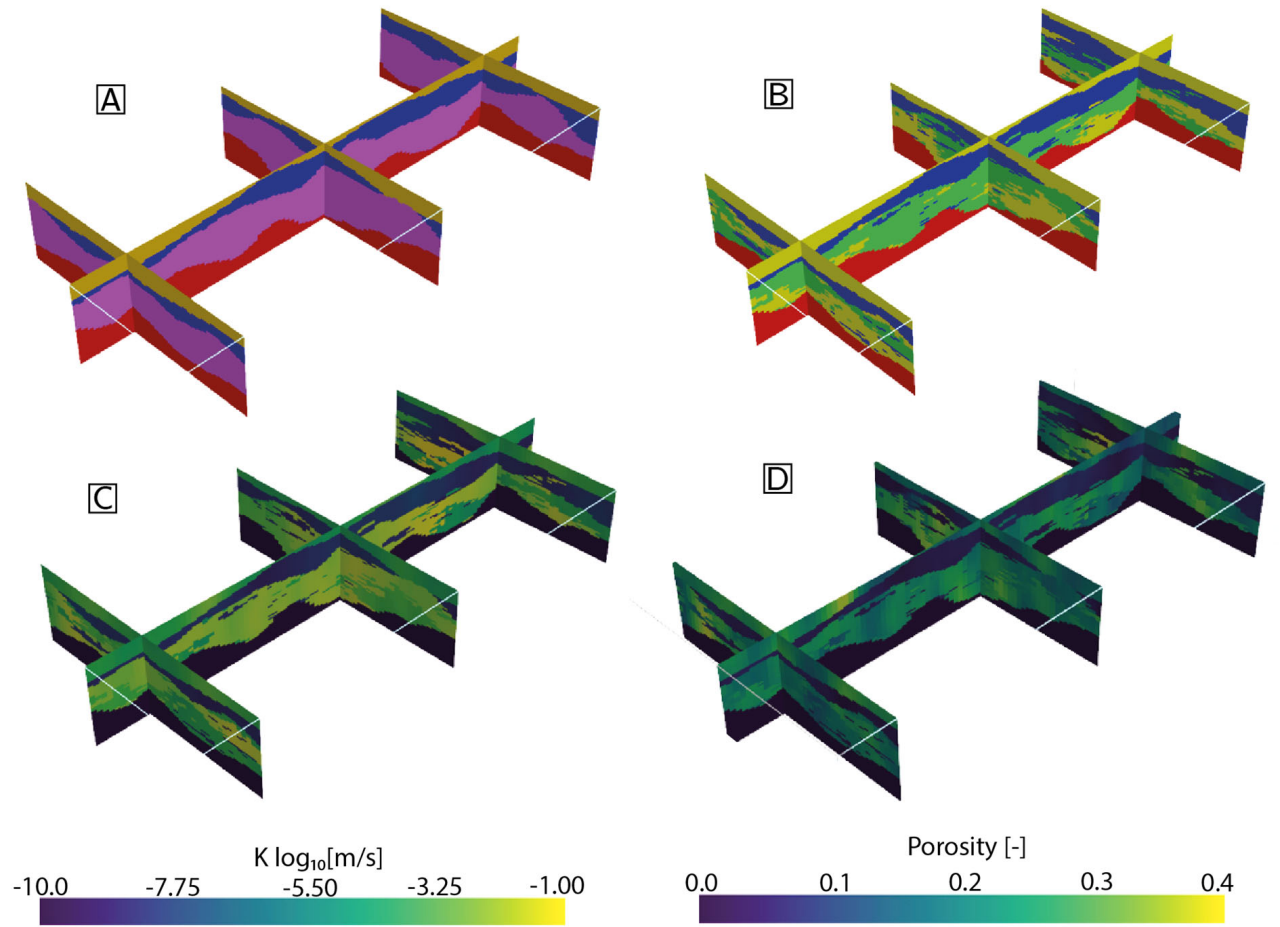
Detailed ArchPy model used for the example. (A) Stratigraphic units, see Figure 3A for the color legend. (B) Facies model, see Figure 3B for the color legend. (C) Hydraulic conductivity in $\log_{10}\left[\frac{m}{s}\right]$. (D) Porosity.

Figure 4 shows the resulting geological model generated by ArchPy using the pile shown in Figure 3. The four stratigraphic units are clearly identifiable (Figure 4A), and the two aquifers (units D and B) are generally separated by unit C, which is composed of clays. In some locations, a direct connection between units D and B can be observed. Heterogeneous facies are simulated in unit B (Figure 4B): relatively large sand patches are present in a gravel‐dominated formation with some small clay occlusions.

The property models for the hydraulic conductivity K (Figure 4C) and porosity (Figure 4D) reflect the heterogeneity present in the units and facies models plus additional small‐scale heterogeneity within the facies. The final property models therefore exhibit several levels and scales of heterogeneity.

### Upscaled Geological Models

To illustrate the impact of the heterogeneity and the effect of choosing a certain grid mode, we compare six different configurations with four different grids.

The *Ap‐he* model corresponds to the fine‐scale original heterogeneous ArchPy model shown in Figure 4. The Ap grid is defined as the original ArchPy grid. It contains 250,000 cells.

The *Ap‐ho* and *Lay‐ho* models consider homogeneous parameters within the stratigraphic units, but two grids are employed. The *Ap‐ho* model uses the original grid (*ArchPy* grid mode). The *Lay‐ho* model uses the *layers* mode, with one layer for each units A, C, and D, and three layers for unit B. It contains 30,000 cells. For these two models, the values of the properties within each unit are given in Table 1. The porosity is equal to 0.2 for all units. It approximately corresponds to the global mean value of the heterogeneous model. For the hydraulic conductivity, $\log_{10}\left(K\right)$ is set to the mean of the underlying Gaussian distributions when a single lithology is present within a unit. For unit B, $\log_{10}\left(K\right)$ is set to the arithmetic mean of the $\log_{10}\left(K\right)$ values for each facies weighted by the proportion of the facies.

**Table 1:** Property Values Used for the Homogeneous Models, Ap‐ho and Lay‐ho, in the Different Stratigraphic Units

Unit domain	*K* $\log_{10}\left[\frac{m}{s}\right]$	Porosity (—)
D	−3.5	0.2
C	−8	0.2
B	−2.3	0.2
A	−10	0.2

The next three models consider heterogeneous parameters computed using the upscaling module Uppy.

The *Lay‐he* model considers the layered grid (identical to Lay‐ho). The upscaled properties are computed from the heterogeneous ArchPy outputs.

The *Nr‐he* model uses the New resolution mode with a factor four in the x direction and two in the y and z directions, resulting in a total 15,625 cells.

**Figure 5:**
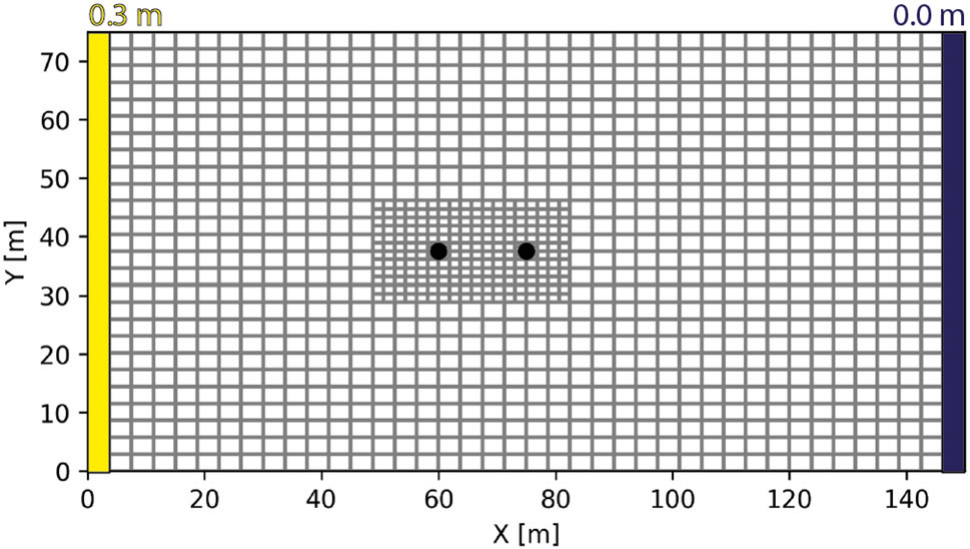
Modflow grid used with the MODFLOW grid mode. The two black dots show the position of the two wells. The other boundary conditions are also shown.

Finally, the *Mg‐he* model uses an existing unstructured MODFLOW grid (Figure 5) with a fine resolution of 3.75 × 3 × 0.75 m (sx×sy×sz), around the two wells and coarse resolution elsewhere. Vertically, the grid contains 10 layers of equal thickness. The grid contains a total of 12,500 cells.

### From ArchPy to MODFLOW

The following code snippet shows how to link ArchPy and Modflow (Listing 1).


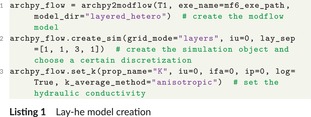


An archpy2modflow object is first created from an existing ArchPy object (T1) by providing only two inputs: the path to the MODFLOW 6 executable (*exe_name*) and the path to the modflow directory (*model_dir*). When creating the modflow simulation (second line), one has to define the *grid_mode*, which is set to “layers” here, the unit realization index (*iu*), which specifies which specific unit realization must be used for the delimitation of the layers, and the *lay_sep* parameter, which specifies how many sublayers must be created within a layer. Finally, for setting up the hydraulic conductivity (third line), one has to indicate the property name corresponding to K in the ArchPy model (prop_name), the units, facies and property realization indices (*iu*, *ifa* and *ip*) to indicate which ArchPy realization must be considered. One has also to indicate if the property is log‐normally distributed (*log*) and how to vertically upscale the cells (*k_average_method*). More information can be found in the ArchPy documentation and examples.^4^ Once these lines of code have been run, the MODFLOW model can be constructed and updated as you would normally do with FloPy, such as adding boundary conditions like rivers, recharge, inflows and outflows from boundaries, etc. It is also possible to modify simulation parameters such as time discretization to make the simulation transient (TDIS package) or solver parameters and criteria (IMS package) if the model is too complex to converge using the default solver. Since the simulation and groundwater flow model can be easily retrieved from the archpy2modflow object, any FloPy modification can be made to the existing model, ensuring flexibility and maximum compatibility with FloPy.

### Groundwater Flow Model

Regarding the hydrogeological context of the example model, a regional hydraulic gradient was defined with a prescribed constant head of 0.3 m on the left side of the model (x=0 m) and 0 m on the right side (x=150 m). A doublet is considered. Groundwater is extracted at a fixed pumping rate ($Q=0.0015\;m^{3}/s$) at position (60.0,25.0,4.5). This water is injected in the aquifer at the same rate at position (75.0,25.0,4.5). The flow is assumed to be in steady state.

With these boundary conditions, MODFLOW 6 is used to solve the groundwater flow for the six different models described above. We then compare the head distributions computed for the six models within the domain. The Ap‐he model is the reference.

**Figure 6:**
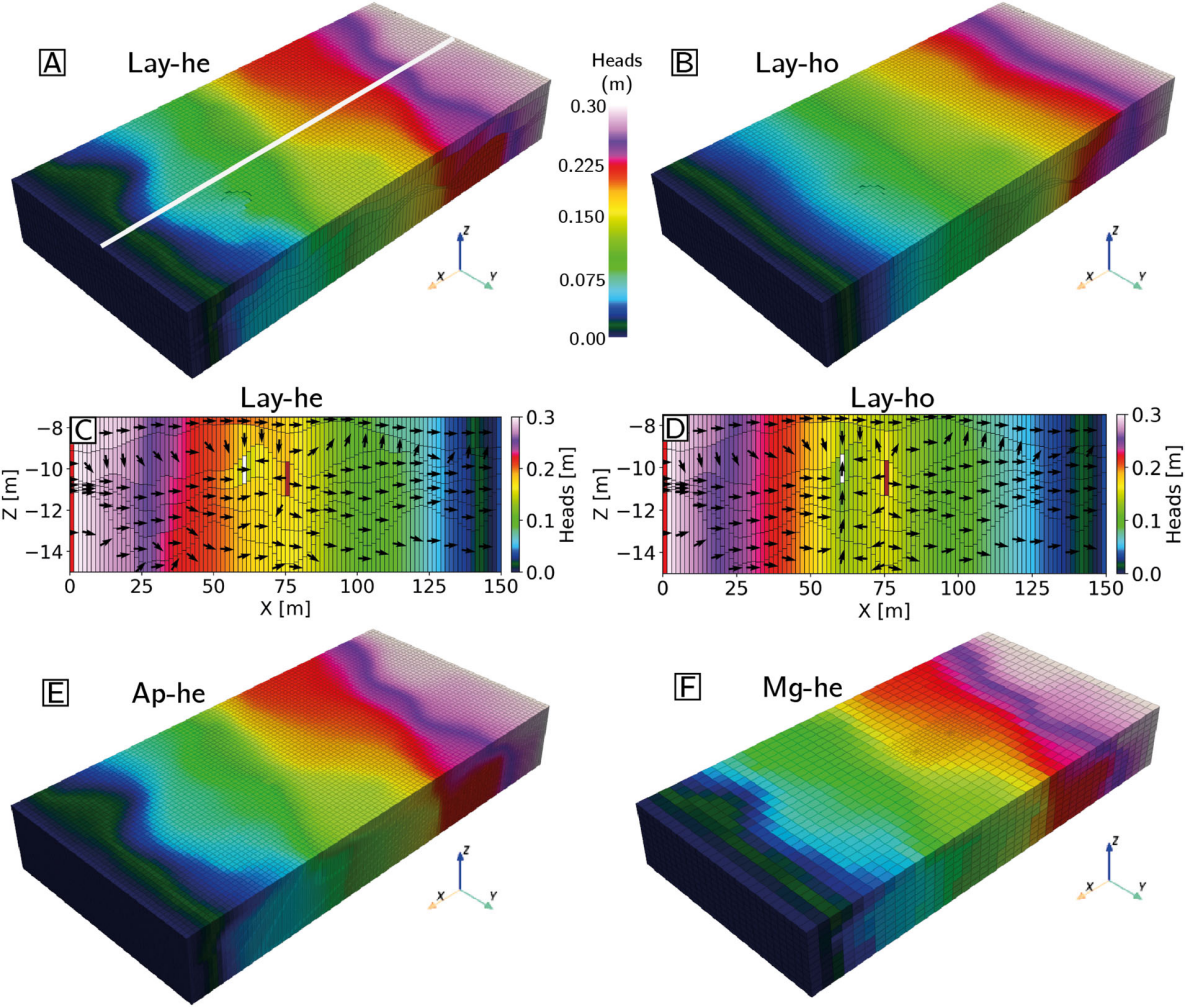
Groundwater flow results for the first example. (A, B, E, F) 3D blocks of the simulated hydraulic heads for Lay‐he, Lay‐ho, Ap‐he, and Mg‐he, respectively. The colorbar shown in (A) is the same for all 3D models. (C, D) Vertical slice views in the 3D blocks at the location of the white line. White and brown cells show the location of the pumping and injecting wells. Red and blue cells show the location of the lateral boundary conditions.

To get a first visual impression of the results, Figure 6 shows the head distributions for the Lay‐he, Lay‐ho, Ap‐he (reference) and Mg‐he models. A rapid inspection indicates that the 3D head fields look globally similar (Figure 6A, 6B, 6E, and 6F), even if the heterogeneous models show a higher variation. It can be noted that the three heterogeneous models (Figure 6A, 6E, and 6F) show a very similar distribution of the heads, compared to the homogeneous model (Figure 6B), indicating the impact of heterogeneity on groundwater flow. Indeed, significant differences can be observed in the cross section of the Lay‐he and Lay‐ho models passing through the center of the model (Figure 6C and 6D). In particular, the influence of the pumping and injection wells is greater in the homogeneous model than in its heterogeneous counterpart. Indeed, the hydraulic heads are lower at the pumping well location in the homogeneous model than in the heterogeneous model, leading to a more pronounced reversal of the hydraulic gradient between the two wells (right to left), compared to the global flow (left to right). This results in a better connection between the two wells. This is probably due to the slightly lower hydraulic conductivity value in unit B in the homogeneous model compared to the heterogeneous model at wells location. Indeed, in heterogeneous model hydraulic conductivity values range from −3 to −1 $\log_{10}\left[\frac{m}{s}\right]$, while, in the homogeneous model, all the cells in this unit have the same conductivity value, −2.3 $\log_{10}\left[\frac{m}{s}\right]$. This value is slightly lower than the conductivity in gravel, to account for the sand and clay facies, that are present in this unit.

A more global and quantitative analysis of the results can be conducted by comparing the simulated heads and groundwater fluxes.

As the different models have different resolutions and grids, it was necessary to average the reference heads (Ap‐he) on all the upscaled cells of the different upscaled grids before comparing them.

**Figure 7:**
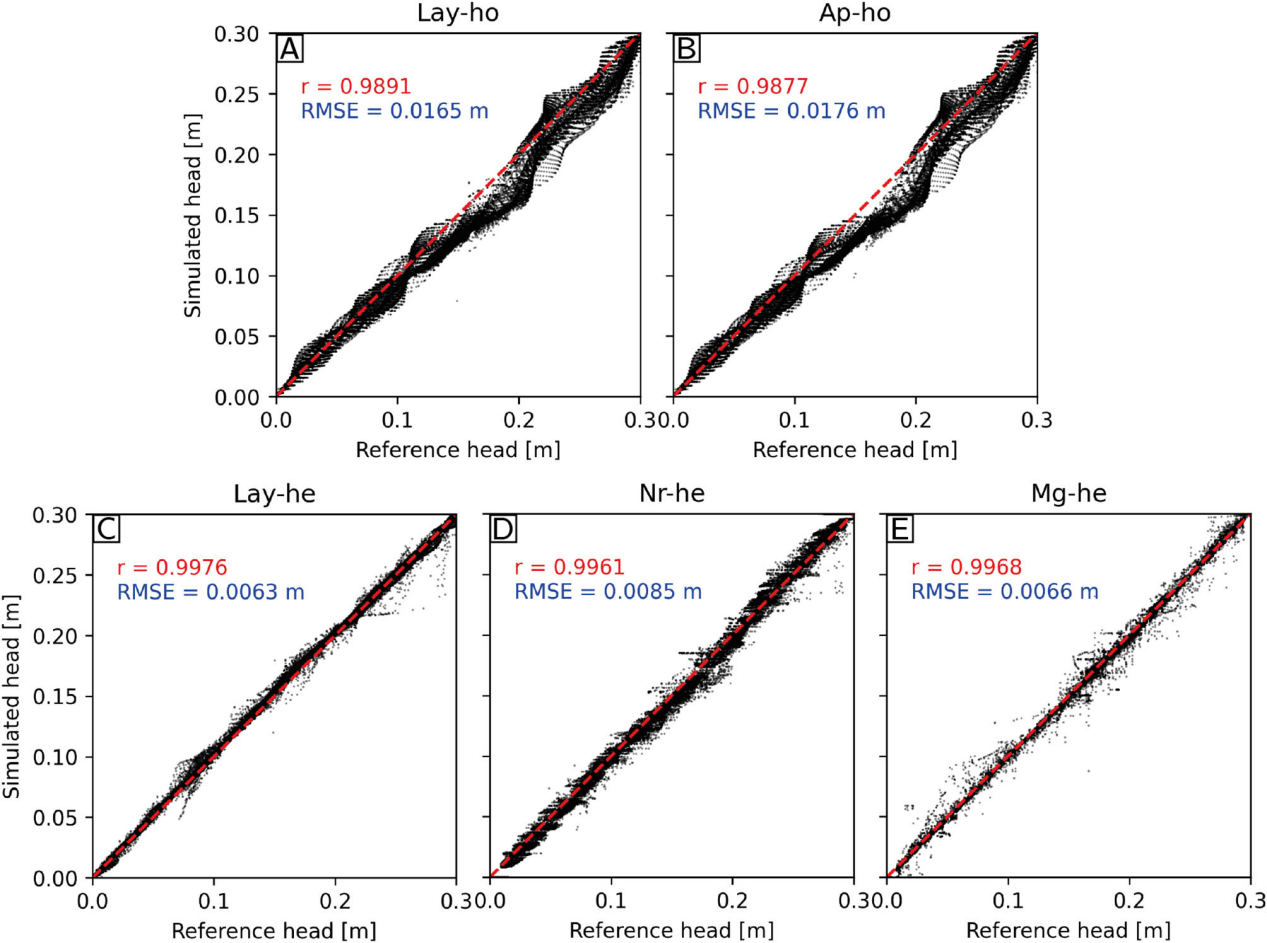
Scatter plots of the simulated heads in Lay‐ho (A), Ap‐ho (B), Lay‐he (C), Nr‐he (D), and Mg‐he (E) models versus the reference head (Ap‐he).

Figure 7 shows the comparison of the reference heads with the heads simulated with the different upscaling modes. The Lay‐he, Nr‐he, and Mg‐he models better reproduce the groundwater heads of the reference than the homogeneous models (Ap‐ho and Lay‐ho). The root mean square error (RMSE) of the Lay‐he model is the smallest, with a value of 0.0063 m (Figure 7C), while it is maximal for Ap‐ho with a value of 0.0176 m (Figure 7B). Overall, the Lay‐he and Mg‐he models give the best results.

Table 2 compares the Darcy flux vectors of the different models with the reference. Again, it was necessary to average the fluxes from the Ap‐he model to match the resolution of the other grids. The vectors were compared in terms of Euclidean distance and cosine similarity. We also compared the total outflowing flux from the models and the reference. The first observation that one can make from Table 2 is that the ranking of the methods differs for the different metrics. Globally, the heterogeneous models perform better than the homogeneous ones. For this example, the model Lay‐he provides the most accurate estimation of the groundwater flow directions. However, the same model Lay‐he shows a rather important error (8.3%) for the total outflow (Column 3). Mg‐He is the model that is globally the best because it has a low error on the total outflow and reproduces the details of the local flux vectors better than most of the other models.

**Table 2:** Measure of Groundwater Flow Similarity Between the Reference (Ap‐he) and the Other Models

Models	Flux Vectors: Distance $\left(m^{3}/s\right)$	Flux Vectors: Cosine Sim. (—)	Total Outflowing Flow Error (%)
Lay‐he	$1.6\cdot 10^{-6}$	0.95	8.3
Mg‐he	$3.4\cdot 10^{-6}$	0.87	−0.2
Nr‐he	$2.0\cdot 10^{-6}$	0.80	−11.1
Ap‐ho	$4.2\cdot 10^{-6}$	0.86	−2.5
Lay‐ho	$4.3\cdot 10^{-6}$	0.86	0.5

Note: Three metrics are used: the Euclidean distance (first column) and the cosine similarity (second column) on the groundwater vector flux in each cell, and the total discharge error compared to the Ap‐he model expressed in % (third column).

### Particle Models

Another novel feature of ArchPy2Modflow is the possibility to create a PRT model to track advective pathways. The user must provide the initial particle locations and the name of the property corresponding to the effective porosity. Additional information can be supplied if available such as different releasing times for the particles.

To illustrate this feature, 2000 particles were randomly drawn within specified bounds using uniform distributions. The coordinates of the starting location of the particle $\left(x_{p},y_{p},z_{p}\right)$ are specified as follows: $x_{p}\in \left[1,20\right]$, $y_{p}\in \left[15,60\right]$, and $z_{p}\in \left[-11,-9\right]$. This area corresponds to the upstream part of the model. Particles were directly released at the beginning of the simulation and were only stopped when reaching a boundary condition, either the pumping well or the downstream boundary. The PRT was performed on the six models but only results from Ap‐he and Lay‐he are shown for simplicity. For each of the six models, the same 2000 particles were used.

**Figure 8:**
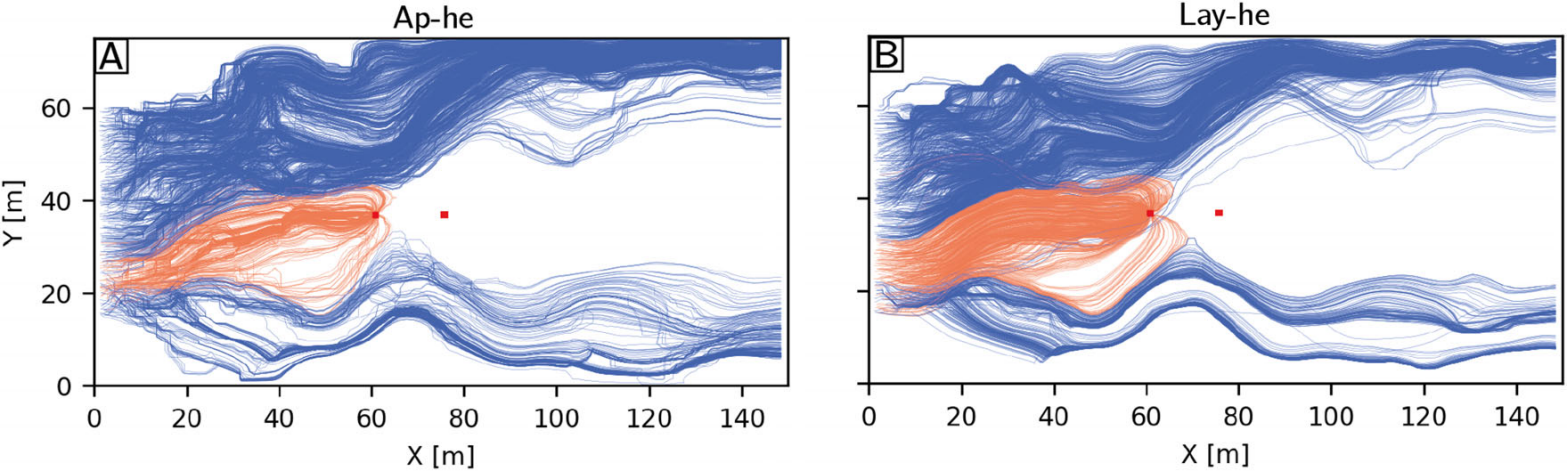
2D projection of particle trajectories in the horizontal plane for Ap‐he model (A) and Lay‐he model (B). The locations of the pumping and injection wells are shown as two red dots. The difference in color of the particle paths indicates whether a particle is captured by the pumping well: blue indicates that the particle exits in the downstream boundary condition; orange indicates that the particle exits in the well.

Figure 8 shows the pathlines of the particles projected on the horizontal xy plane for Ap‐he (Figure 8A) and Lay‐he models (Figure 8B). Globally, we can distinguish two groups of particles, those that end up in the well (orange lines in Figure 8) and those that end up in the downstream boundary condition (blue lines in Figure 8). Both models show similar patterns for the pathlines, indicating that the upscaled model (Lay‐he) reproduces rather accurately the detailed and intricate particle paths.

**Figure 9:**
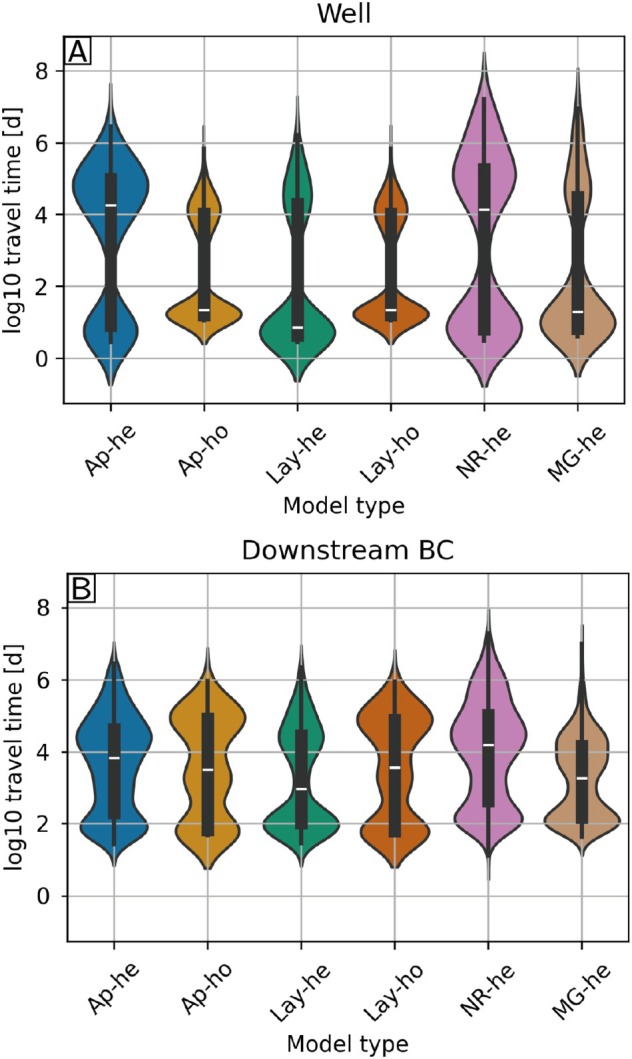
Statistical distributions of the arrival time at the two types of outlets: the pumping well (A) and the downstream boundary (B) for each of the six models. The horizontal white line represents the median of the distribution.

We also compared the statistics of the arrival times of the particles at the different outlets for the six models (Figure 9). The distributions of the arrival times at the pumping well (Figure 9A) show two modes corresponding to two groups of particles representative of the initial lithology in which the particles were released. The particles released in permeable media (sand, gravel) have short arrival times ($10^{0}-10^{2}$ days) and those released in low permeability media (clay) have longer arrival times ($10^{4}-10^{7}$ days). The two homogeneous models perform similarly and are quite different from the reference (Ap‐he). For the heterogeneous models, Ap‐he and Nr‐he show a high proportion of particles in the late group (50–70%), while it is the opposite for the Lay‐he and MG‐he models (20–30%). Regarding the downstream boundary conditions (Figure 9B), the distributions are more uniform with values between $10^{2}$ and $10^{7}$ days. However, we still observe that Ap‐he and Nr‐he show later arrival times with a higher median compared to the other models, and in particular Lay‐he and Mg‐he. This rapid comparison shows that, if we only consider the arrival time distribution, Nr‐he seems to be the closest to the reference for this particular example.

Finally, it is interesting to compare the computation time of the different steps of the modeling workflow for the different variants. The results are shown in Table 3. We see that the computation time, both for flow and PRT, increases with the number of cells as expected. Heterogeneity has also an important impact on computation times: solving Ap‐he is nearly two times slower than Ap‐ho while the resolution is identical. Nr‐he and Mg‐he show the fastest flow computation but with the disadvantage of a larger time required for the upscaling, in particular for the Mg‐he model. This is due to the UG, which requires identifying which fine cells fall into which coarse cell. It should be noted that the upscaling methods implemented in Uppy have not yet been optimized, and this will be the task of future work. If we consider the problem globally, the choice of the grid mode is a trade‐off between the required level of accuracy and details, and the available computing time and resources. In the example that we show here, the best trade‐off is obtained with the Lay‐he model, which has a good accuracy and a small total computing time.

**Table 3:** Computational and Upscaling Time for the Different Grid Modes.

			Comp. Time	Upsc.	Total
Models	Number of Cells	Comp. Time Flow (s)	PRT (s)	time (s)	time (s)
Ap‐he	250,000	9.88	5.89	0	15.77
Ap‐ho	250,000	4.89	3.10	0	7.99
Lay‐he	30,000	0.56	1.78	0.9	3.24
Lay‐ho	30,000	0.49	1.78	0	2.3
Nr‐he	15,625	0.41	1.88	6.0	8.29
Mg‐he	12,500	0.32	2.30	11.9	14.52

Note: The tests were made on a Windows computer equipped with an Intel Core i7‐7700 (3.60 GHz). All simulations were performed on a single thread.

It is important to note that these results have been obtained in steady‐state. The computational gain from upscaling is likely greater when the flow regime is transient, and simulation time may be much longer. These considerations are also very important when the parameters have to be identified using an optimization problem or an ensemble approach. Furthermore, models with fewer cells require less memory, which may also be an advantage when a large number of models need to be run in parallel. Thus, the choice of a particular grid mode depends heavily on the questions that the model is intended to answer.

## Discussion

A major advantage of the tools presented in this paper is that they facilitate the comparison of different conceptual models and upscaling strategies. In a situation where the choice of the parameters defining a conceptual model is difficult, different SPs encompassing the various plausible sedimentological settings (e.g., for a given unit) can be created. Then the resulting ArchPy models can be used as input in a groundwater model, and the results compared with real groundwater measurement data to determine the most suitable SP. Other kinds of data, such as geophysical data, could also be considered.

Overall, the ArchPy2modflow and Uppy tools can be used in a wide range of geological contexts, but they also have some limitations. Currently, ArchPy can only model geological environments that are not heavily impacted by tectonics. It cannot model faults or overturned folds because it was designed to model sedimentary Quaternary environments and to generate as rapidly as possible an ensemble of stochastic realizations. But ArchPy integrates several advanced geostatistical methods such as Multiple Point Statistics (Mariethoz et al. 2010; Dall'Alba et al. 2020). Using appropriate parameters and datasets, it can represent a wide range of complex sedimentological settings such as channels, lobes, lenses, or gravel beds.

Uppy can upscale any field of continuous values, as long as the input grid is regular. If the simulated geological structures are very thin, tortuous, and connected, care must be taken to use a grid that ensures that these structures are accurately preserved. One can, for example, refine locally the simulation grid along these structures. One limitation of Uppy is that it is implemented in native Python, which is less numerically efficient than if it were written in a compiled language such as C or C++. Accelerating the upscaling may be the focus of future developments.

Another important aspect is the integration of ArchPy with inverse methods for groundwater model calibration. Using the tools presented in this paper, it would be possible to develop a full inverse framework that has the potential to preserve geological consistency when fitting a groundwater model to measurement data. For example, in a previous work, we showed that it was possible to couple ArchPy and forward geophysical and hydrogeological simulators to infer the geometry of the sedimentary units and the spatial distribution of the petrophysical parameters within the units Neven and Renard (2023). This highlights that it would be particularly interesting to couple the new tools presented in this paper with parameter estimation software, such as PEST and PEST++ (Doherty and Simmons 2013; White et al. 2020). Geological parameters in ArchPy, such as variograms, or ArchPy outputs, such as geological surfaces, could be treated as PEST parameters by calibrating a groundwater model. Different research directions are possible, such as employing ensemble‐based techniques (Lam et al. 2020), traveling pilot points (Khambhammettu et al. 2020), or particle filters (Ramgraber et al. 2020). But it remains unclear which inverse methods will be the most efficient and reliable (Juda et al. 2023) for these complex models. One promising technique could be to use an ensemble of ArchPy models and forward simulator predictions to learn the statistical relations between data, parameters, and forecasts. These relations could then be used to produce direct forecasts conditional on observed measurements (Scheidt et al. 2015; Sun and Durlofsky 2017; Delottier et al. 2023).

## Conclusion

This paper introduced two new tools, Archpy2Modflow and Uppy, that allow a straightforward integration of aquifer heterogeneity into groundwater models. Uppy allows for upscaling fine and detailed property models into coarser grids. Four different options are available depending on the user's needs, including the possibility to directly import an existing MODFLOW grid. These four different options provide a wide range of possibilities to meet possible user needs and adapt to different problems. ArchPy2Modflow, using Uppy, makes the link between the stochastic geomodeling python library ArchPy and MODFLOW 6, enabling the easy assignment of the geological properties to MODFLOW parameters. As of today, ArchPy2Modflow directly supports the integration of properties for the main MODFLOW 6 models, which are groundwater flow, transport, energy, and PRT models. It is important to note that ArchPy2Modflow and FloPy are complementary. The models created with ArchPy can be enriched and improved directly with FloPy. If a MODFLOW/FloPy module has not been implemented within Archpy2Modflow, it is possible to incorporate it with FloPy.

Overall, Archpy2Modflow is part of an effort to facilitate the combination of open source solutions in the field of geosciences and hydrogeology, paving the way for faster, easier, and more reproducible integration of heterogeneity, ultimately leading to better consideration of the uncertainty.

## Author's Note

The authors declare no potential conflict of interest.

## Data Availability

The github repository of the code can be found on the github repository of ArchPy: https://github.com/randlab/ArchPy/tree/main/. The synthetical dataset and codes for the synthetic example can be found on https://github.com/randlab/ArchPy/tree/main/examples/14_archpy2modflow_paper.

## Footnotes

^1^

https://github.com/randlab/ArchPy.

^2^

https://archpy.readthedocs.io/.

^3^

https://github.com/randlab/ArchPy/blob/main/examples/14_archpy2modflow_paper/ap2_mf_paper.ipynb.

^4^

https://archpy.readthedocs.io/.

## Modeling Parameters

Table A1 shows the variograms used for each unit to construct the unit model.

**Table A1:** Covariance Models Parameters (*C*: Contribution, i.e., Sill; *r*: Range) Used for the Surfaces Interpolation of Each Top Surface of the Units

Units	*r* ^cub^ (m)	*C* ^cub^ (m^2^)
C	(40, 40)	0.2
B	(30, 30)	0.6
A	(30, 30)	0.6

Noe: All models are isotropic. Ranges are indicated in the two main directions: *x* and *y*. Subscript *cub* indicates cubic model, which is a specific type of covariance model.

Table A2 shows the variograms used to simulate the facies within unit B with the SIS method.

**Table A2:** Covariance Models Parameters (*C*: Contribution, i.e., Sill; *r*: Range) Used for the Sequential Indicator Simulation in Unit B

Facies	*r* ^sph^ (m)	*C* ^sph^ (m^2^)
Gravel	(20, 20, 2)	0.24
Sand	(20, 20, 2)	0.21
Clay	(30, 30, 0.5)	0.1

Note: Subscript *sph* indicates spherical model, which is a specific type of covariance model. Ranges are indicated in the three main directions: *x*, *y*, and *z*.

Table A3 shows the variograms used to simulate the two properties, hydraulic conductivity and porosity, within the different facies. We have used the same variogram model for all facies.

**Table A3:** Covariance Models Parameters (*C*: Contribution, i.e., Maximum Variance; *r*: Range) Used for Property Simulations

Property	*r* ^cub^ (m)	*C* ^cub^
Hydraulic conductivity [$\log_{10}\left[\frac{m}{s}\right]$]	(25, 25, 25)	0.25
Void ratio sand [$\log_{10}\left[-\right]$]	(15, 15, 15)	0.005
Void ratio gravel [$\log_{10}\left[-\right]$]	(15, 15, 15)	0.005
Void ratio clay[$\log_{10}\left[-\right]$]	(15, 15, 15)	0.026
Void ratio basement [$\log_{10}\left[-\right]$]	(15, 15, 15)	0.0218

Note: Subscript *cub* indicates cubic model, which is a specific type of covariance model. Ranges are indicated in the three main directions: *x*, *y*, and *z*.
